# Calycosin ameliorates osteoarthritis by regulating the imbalance between chondrocyte synthesis and catabolism

**DOI:** 10.1186/s12906-023-04314-z

**Published:** 2024-01-22

**Authors:** Hong Su, Qiuju Yan, Wei Du, En Hu, Zhaoyu Yang, Wei Zhang, Yusheng Li, Tao Tang, Shushan Zhao, Yang Wang

**Affiliations:** 1 Department of Integrated Traditional Chinese and Western Medicine, Institute of Integrative Medicine, Xiangya Hospital, Central South University, Changsha, 410008 P.R. China; 2 National Clinical Research Center for Geriatric Disorders, Xiangya Hospital, Central South University, Changsha, 410008 Hunan P.R. China; 3 Department of Orthopedics, Xiangya Hospital, Central South University, 87 Xiangya Road, Changsha, 410008 Hunan Province China; 4 Department of Rehabilitation Medicine, Xiangya Hospital, Central South University, 87 Xiangya Road, Changsha, 410008 Hunan Province China; 5 The College of Integrated Traditional Chinese and Western Medicine, Hunan University of Chinese Medicine, Changsha, 410208 China

**Keywords:** Calycosin, Osteoarthritis, Network pharmacology, Molecular docking, Inflammatory, Cyclooxygenase 2

## Abstract

Osteoarthritis (OA) is a severe chronic inflammatory disease. As the main active component of *Astragalus mongholicus Bunge*, a classic traditional ethnic herb, calycosin exhibits anti-inflammatory action and its mechanism of exact targets for OA have yet to be determined. In this study, we established an anterior cruciate ligament transection (ACLT) mouse model. Mice were randomized to sham, OA, and calycosin groups. Cartilage synthesis markers type II collagen (Col-2) and SRY-Box Transcription Factor 9 (Sox-9) increased significantly after calycosin gavage. While cartilage matrix degradation index cyclooxygenase-2 (COX-2), phosphor-epidermal growth factor receptor (p-EGFR), and matrix metalloproteinase-9 (MMP9) expression were decreased. With the help of network pharmacology and molecular docking, these results were confirmed in chondrocyte ADTC5 cells. Our results indicated that the calycosin treatment significantly improved cartilage damage, this was probably attributed to reversing the imbalance between chondrocyte synthesis and catabolism.

**Supplementary Information:**

The online version contains supplementary material available at 10.1186/s12906-023-04314-z.

## Introduction

Osteoarthritis (OA) is a severe, chronic inflammatory disease [1], which is estimated to affect more than 528 million people worldwide by 2010 [2]. With the accelerated aging of the world population, the social and economic burden brought by OA is increasing year by year [3, 4].

OA is characterized by progressive cartilage degeneration. Chondrocytes are the only cell types in cartilage that function in the synthesis and catabolism of the extracellular matrix (ECM). The diminished cartilage matrix is primarily contributed to the decreased secretion of type II collagen (Col-2) by chondrocytes, whose transcription is controlled by SRY-Box Transcription Factor 9 (Sox-9) [5–8]. As an important local source to produce inflammatory mediators, chondrocytes will activate cycloxygenase-2 (COX-2) and matrix metalloproteinases (MMPs) expression [9]. These inflammatory mediators further affect chondrocytes and stimulate inflammatory cytokines, such as tumor necrosis factor- α (TNF-α), and interleukin (IL)-1β [10, 11], which ultimately lead to cartilage degradation. Moreover, the lubrication of joint synovial fluid also has an important role in cartilage injury. Recent studies revealed that the importance of epidermal growth factor receptor (EGFR) Signaling in OA. For example, cartilage-specific inactivation of EGFR in mice leads to fewer superficial chondrocytes [12, 13]. Two EGFR ligands were found to be higher than normal in human OA samples [transforming growth factor alpha (TGFα) and Heparin-binding epidermal growth factor-like growth factor (HB-EGF)] were found to be higher than normal in human OA samples [14, 15]. Two genome-wide association studies (GWAS) revealed that TGF is one of the most associated genes with human OA [16], which stimulated MMPs expression [17]. These studies strongly suggest that EGFR signaling is involved in OA pathology.

Nonsteroidal anti-inflammatory drugs (NSAIDs) are currently the main drug for OA [18]. However, the clinical use of NSAIDs should be aware of gastrointestinal and cardiovascular adverse events. Furthermore, there are patients with contraindications or no response to NSAIDs [19]. Selective COX-2 inhibitors can reduce the incidence of gastrointestinal adverse events while exerting significant clinical efficacy [18]. Evidence suggests that inhibition of COX-2 exerts analgesic and anti-inflammatory effects [20]. Although a study has shown that selective COX-2 inhibitors increase adverse outcomes compared with placebo, this adverse event was less than that of NSAIDs [21]. This phenomenon is probably related to these sulfonamide-containing inhibitors that can also inhibit carbonic anhydrase II [22]. Therefore, developing alternative COX-2/EGFR inhibitors that do not disrupt the balance of cartilage synthesis and catabolism is desirable.

*Astragalus mongholicus Bunge* is a classic traditional ethnic herb, mainly distributed in Inner Mongolia, Northeast of Heilongjiang province, China [23]. The most traditional use of *Astragalus mongholicus Bunge* is benefit Qi, which also named “Yiqi” in traditional Chinese medicine (TCM) [24, 25]. Calycosin (7,3ʹ-dihydroxy-4ʹ-methoxy isoflavone, C16H12O5), with PubChem CID: 5,280,448, is the main active component of *Astragalus mongholicus Bunge* [26], which exhibits a variety of pharmacological effects, including anti-inflammatory [27, 28], anti-oxidant [29] and anti-osteoporotic [30] activities. The Chinese traditional herb *Astragalus mongholicus Bunge* (huáng qí) has been effective in treating many diseases, including OA [31, 32]. Calycosin has been shown to inhibit NF-κB activation and reduce the production of pro-inflammatory cytokines, such as TNF-α, IL-1β, and IL-6 [27, 28]. Furthermore, it has been reported to reduce the expression of MMPs (especially MMP3 and MMP13), which are essential for the degradation of cartilage matrix [33, 34]. A Molecular Dynamics simulation suggested that calycosin may bind to the Interleukin-6 receptor (IL-6R) [35]. Additionally, calycosin shows a similar structure to estrogen estradiol and acts as a plant-derived phytoestrogen. Thus, it possesses estrogen-like activity, such as preventing subchondral bone resorption and cartilage degradation [36]. It has been reported that calycosin-7-O-beta-D-glucopyranoside can reduce the severity of OA-like structural damages in the cartilage, although it does not affect PGE2 expression [37]. Consequently, calycosin may be a potential drug for the prevention and treatment of OA. However, its mechanism of action and targets have not yet been determined.

TCM is a complex holistic healthcare system that emphasizes a balance between body, mind, and environment. Currently, there has been an increasing interest in the scientific validation of TCM therapies and the development of new drugs from natural products [38, 39]. Network pharmacology is an emerging field that combines systems biology and bioinformatics, providing a powerful tool for studying the complex interactions between multiple targets and pathways involved in TCM therapies [40].

**Fig. 1:**
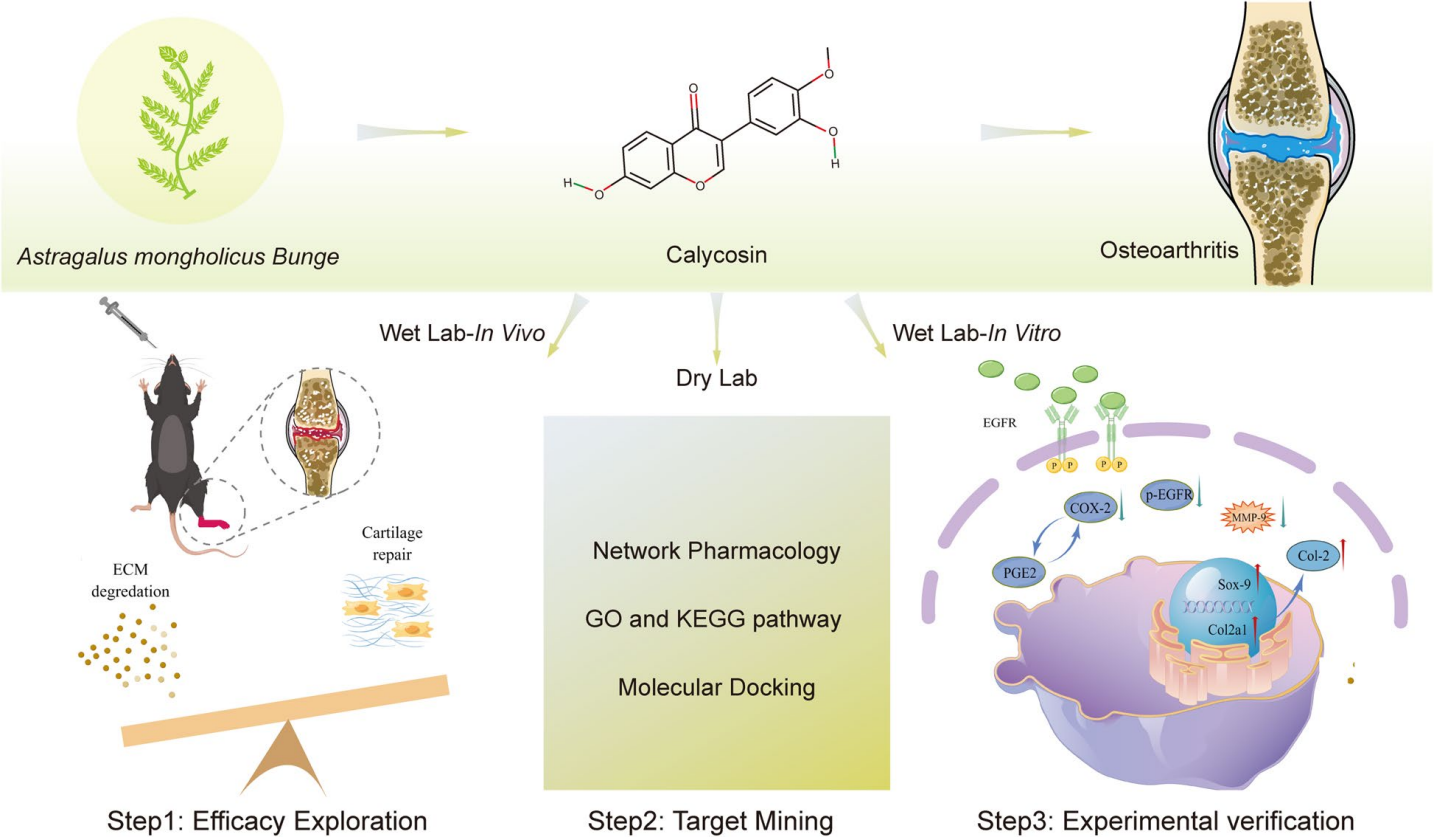
Graphical abstract

In the current study, we evaluated the effect of calycosin on OA in vivo and in vitro. Firstly, we investigated the effects of calycosin on articular cartilage under OA conditions in vivo. We then utilized computational tools and resources (Dry Lab) to investigate the pharmacological network and predict potential targets [41]. Finally, in vitro experiments (Wet Lab) were conducted to explore the underlying mechanisms (Fig. 1).

## Materials and methods

This work adopts a recommended research approach and writing logic [41, 42]. Combining bioinformatics mining and experimental verification, the study consists of a total of two main parts- Dry Lab and Wet Lab.

### Dry Lab

#### Prediction of potential targets for calycosin

The potential targets of calycosin were gathered from Similarity Ensemble Approach (SEA, http://sea.bkslab.org/) [43, 44], Swiss Target Prediction (https://www.Swiss.target.prediction.ch/) [45], PharmMapper (http://lilab-ecust.cn/pharmmapper/), STITCH (http://stitch.embl.de/) [46], and Traditional Chinese Medicine Systems Pharmacology (TCMSP, http://lsp.nwu.edu.cn/tcmsp.php) [47] databases and analysis Platform, where “Homo Sapiens” were selected as targeted species.

#### Collection of Gene Targets for OA

Using “Osteoarthritis “, Therapeutic gene targets of OA were collected from the therapeutic target database (TTD, https://db.idrblab.org/ttd/, September 29th, 2021) [48], Drug Bank (http://www.drugbank.ca/) [49], Online Mendelian Inheritance in Man (OMIM, https://www.ncbi.nlm.nih.gov/omim/) [50], and Gene Cards (http://www.genecards.org/, Version 5.9) [51].

#### Construction of protein interaction networks

The intersection of genes between OA and calycosin produced by all databases was taken for further network analysis. These overlapping genes were uploaded to the Search Tool for Recurring Instances of Neighboring Genes (STRING, http://string905.embl.de/, version11.0b) database [52] to generate protein–protein interaction (PPI) networks using “Homo sapiens” as the background (combined score > 0.4, medium confidence). Subsequently, we applied Cytoscape visualization software 3.7.2 (http://www.cytoscape. org/) to evaluate PPI networks, the Molecular Complex Detection (MCODE, http://apps.cytoscape.org/apps/mcode) and CytoHubba (http://apps.cytoscape.org/apps/cytohubba) plugins were applied to find core clusters and hub genes.

#### GO and KEGG pathway enrichment analysis

The Database for Annotation, Visualization, and Integrated Discovery (DAVID, https://davidd.ncifcrf.gov/) [53] version 6.8 (background and species = Homo sapiens) was used to perform GO and KEGG pathway enrichment analysis [54]. A P < 0.01 was considered to indicate a statistically significant difference. Sankey diagram and dot plot were created by http-s://www.bioinformatics.com.cn, a free online platform for data analysis and visualization.

#### Molecular docking

The three-dimensional structure of calycosin was obtained from PubChem (https://pubchem.ncbi.nlm.nih.gov/). PDB files of key proteins from PPI were searched and downloaded from Universal Protein Resource (UniProt, https://www.uniprot.org/) [55] and Protein Data Bank (PDB: http://www.rcsb.org/pdb) databases [56, 57], respectively. Next, with the help of AutoDockTools (version 1.5.6), we completed ligand preparation by removing water, and adding hydrogens and charges. AutoDock Vina (version 1.1.2) [58] was used for molecular docking (energy range = 4, exhaustiveness = 8) with the same box size (size x = 40, size y = 40, size z = 40). Finally, we use PyMoL (version 2.6) [59] and BIOVIA Discovery Studio Visualizer (version 4.1.0) [60] after molecular docking for graphical visualization. The biographical tools and website information used in this article are summarized in Table 1.

**Table 1:** Overview of bioinformatic tools used in this article

Functions	Tools	Websites
Prediction of potential targets for Calycosin	Similarity Ensemble Approach (SEA)	http://sea.bkslab.org/
	Pharm Mapper	http://lilab-ecust.cn/pharmmapper/
	STITCH	http://stitch.embl.de/
	Traditional Chinese Medicine Systems Pharmacology (TCMSP)	http://lsp.nwu.edu.cn/tcmsp.php
Collection of Gene Targets for OA	therapeutic target database (TTD)	https://db.idrblab.org/ttd/, September 29th, 2021
	Drug Bank	http://www.drugbank.ca/
	Online Mendelian Inheritance in Man (OMIM)	https://www.ncbi.nlm.nih.gov/omim/
	Gene Cards	http://www.genecards.org/
Construction of PPI	Search Tool for Recurring Instances of Neighboring Genes (STRING)	http://string905.embl.de/, version11.0b
GO and KEGG Pathway Analysis	Database for Annotation, Visualization, and Integrated Discovery (DAVID)	https://davidd.ncifcrf.gov/
Molecular Docking	PubChem	https://pubchem.ncbi.nlm.nih.gov/
	Universal Protein Resource (UniProt)	https://www.uniprot.org/
	Protein Data Bank (PDB)	http://www.rcsb.org/pdb

### Wet Lab

#### Animals

The animal protocols were approved by the ethics Committee on the Use and Care of Animals of Central South University and were cared in the Experimental Animal Center of Xiangya Hospital (NO.202103631). Male C57BL/6 mice were housed in identical conditions (12 h light–dark cycle, 23 ± 2℃, a relative humidity of 50 ± 10%) in accordance with the Guide for the Care and Use of Laboratory Animals of the National Institutes of Health (NIH Publication No. 85–23, revised 1996).

#### Mice knee OA model and Experimental Design

OA model was induced by anterior cruciate ligament transected (ACLT) [61]. In the sham group, we conducted a simulated operation (open the joint capsule and then suture the incision). However, the bilateral anterior cruciate ligament was transected in OA and calycosin treated groups.

The mice were randomly divided into three groups: the sham group, the OA model group, and the calycosin treatment group (n = 10 per group). Based on previous experience, the calycosin group was treated with calycosin (20,575–57-9, Sinopharm Chemical Reagent Co., Ltd., Shanghai, China) 50 mg/kg once a day by oral gavage [62]. The sham and OA groups received an equivalent volume of physiological saline. The gastrectomy was performed at the same time of the day, and the rest of the diet and water are the same for free. The knee samples were harvested by cardiac perfusion after 4 weeks post-operatively. Animals were anesthetized with 0.3% pentobarbital (0.025 ml/mg) intraperitoneally so that they lost consciousness.

#### Safranin-O/Fast green staining

Mice joint surfaces were fixed overnight with 4% paraformaldehyde, then decalcified in 10% EDTA for 2 weeks, and finally embedded in paraffin. Sections (5 μm) were cut from the paraffin blocks and stained with Safranin O (0.2%)/Fast Green (2%) solution (G1053, Servicebio, Wuhan, China). Osteoarthritis research society international (OARSI) scoring system was used to assess joint pathology [63].

#### Toluidine blue staining

The sample preparation process was the same as above. Differently, the hydrated sections were stained with 0.5% toluidine blue (G1032, Servicebio) for 4 min at 37 °C. The tissues were then differentiated by 0.1% glacial acetic acid and finally sealed for observation. under a Zeiss-Axio Imager M2 fluorescence microscope (Carl Zeiss, Oberkochen, Germany).

#### Cell culture and grouping

The ADTC5 cells (HTX2518, OTWO, Shenzhen, China) were cultured in Dulbecco's Modified Eagle Medium/Nutrient Mixture F-12 (DMEM/F-12) (L320KJ, BasalMedia, Shanghai, China) supplemented with 10% fetal bovine serum (10,270,106, Gibco, CA, USA) and 1% Penicillin–Streptomycin Solution 100 × (SL6040, Coolaber, Beijing, China) at 37 ◦C in 5% CO2 (#3111, Thermo Fisher Scientific, MA, USA).

Cells contain 6 groups: normal control (CON) group, OA model (MOL) group, solvent control (DMSO) group and different dose of calycosin (CAL) groups. In order to simulate the pathological process of aseptic inflammation in chondrocytes ADTC5 cells in vitro, we used IL-1β (prp1119, Abbkine Scientific, Wuhan, China)10 ng/mL as a stimulation factor [64]. The cells were pretreated with different concentrations (0, 16, 32, and 64 µM) of calycosin (A0514, MUST Biotechnology, Chengdu, China) with 10 ng/mL IL-1β for 24 h. DMSO (CD4731C, Coolaber) control was performed at the same concentrations with 32 µM.

#### Protein extraction

Protein extracts were prepared from mid-log phase cells. Briefly, mid-log phase cells were washed with ice-cold Phosphate-buffered saline (PBS, pH = 7.4), then scraped the cells and lysed on ice for 30 min in RIPA-lysis buffer (P0013B, Beyotime Biotechnology, Shanghai, China) supplemented with a complete protease (P1050, Beyotime) and phosphatase (P1051, Beyotime) inhibitor cocktail. Extracts were clarified by centrifugation (12,000r for 20 min at 4 °C) and were stored at − 80 °C until analysis. The BCA assay kit (P0011, Beyotime) was used to determine the protein concentration in each sample according to the manufacturer's instructions.

#### SDS-PAGE and Western blotting

Generally, 20μg of proteins were separated by 8% SDS-PAGE gel electrophoresis and electro-transferred to a PVDF membrane. After blocking in 5% non-fat dry milk in 1 × TBST (10 mmol/L Tris, 150 mmol/L NaCl and 2ml Tween 20 in 2L pure water), the membrane was incubated with primary antibodies overnight and HRP-conjugated secondary antibodies for 1 h. Protein bands were detected using ECL reagent and analyzed using the ChemiDoc XRS + imaging system (Bio-Rad, CA, USA). Western blot data were quantified with Image Lab software for the ChemiDoc XRS system (Bio-Rad). The values for the target protein bands were normalized against GAPDH (#5174, Cell Signaling Technology). Statement: we apologize that unable to provide a full-length image due to technical ignore that the blots were cut before hybridisation with antibodies.

#### Immunofluorescence staining

For Immunofluorescence staining, paraffin-embedded tissue sections were subjected to a heat-mediated antigen retrieval procedure. To be specific, after sections were dried, dewaxed, and hydrated, they were heated to repair antigens. Antigens repair was performed with citric acid buffer (PH6.0) in a 92 °C water bath for 60 min and then restored at room temperature. Following that, tissue sections were incubated overnight with a primary antibody [(Col-2 pAb, #28,459–1-AP, Proteintech, Wuhan, China), (Sox-9 Rabbit mAb, #82,630, Cell Signaling Technology, MA, USA)] at 4 °C. The secondary antibody was then added to the sections, which were incubated for 60 min. The nucleus was counterstained with 75% DAPI (C0060, Solarbio) for 10 min. Finally, anti-fluorescence quencher was applied to seal the sections.

Cells were fixed with 4% paraformaldehyde for 20 min, washed with PBS, permeabilized with 0.1% Triton X-100 in PBS for 10 min, and then blocked with 3% BSA (A8010, Solarbio). The primary antibodies were then incubated overnight in PBS at 4˚C, followed by an hour of incubation with the secondary antibodies at room temperature. The rest of the steps are the same as above.

### Statistical analysis

Statistical analysis was processed with GraphPad Prism 9.2 software. Data were analyzed by using one-way analysis of variance (ANOVA) followed by post hoc Dunnett’s t-test for multiple comparisons and presented as the mean ± SD. A value of P < 0.05 was statistically significant.

## Results

### The contributions of calycosin to OA in vivo

**Fig. 2:**
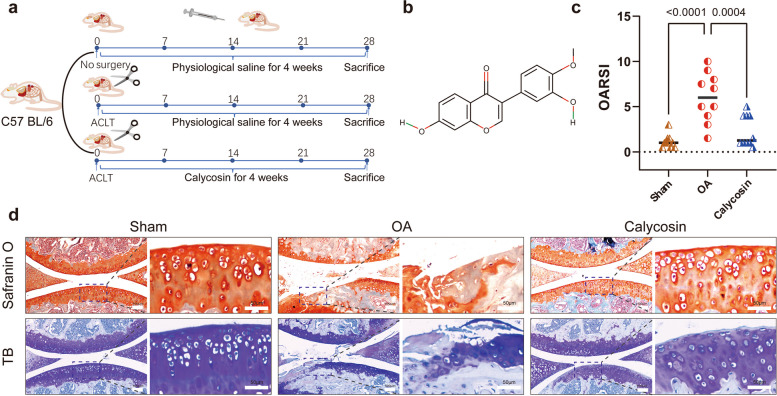
Calycosin alleviates osteoarthritis pathology. **a** Experimental design and animal grouping. **b** Chemical structure of calycosin. **c** OARSI scores. **d** Safranin O/Fast Green and toluidine blue staining showed calycosin reverses cartilage damage after ACLT. N = 10. Data are presented as the mean ± SD

**Fig. 3:**
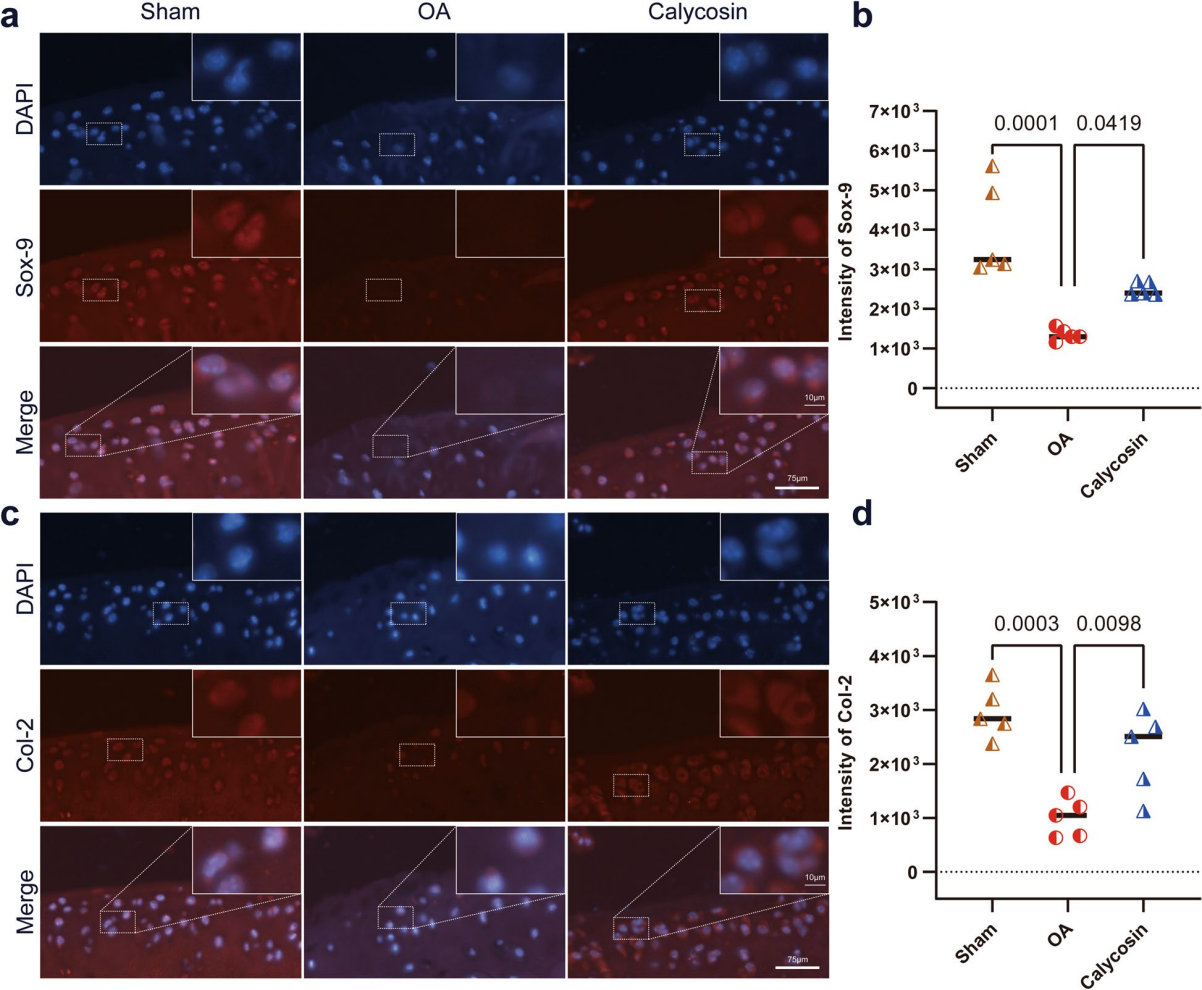
Calycosin contributes to Articular Cartilage Repair. Representative images of immunofluorescence (20X) staining of Sox-9 (**a**) and Col-2 (**c**) in the articular cartilage. DAPI stain was used to label the nucleus. Quantitative analysis of the Sox-9 (**b**) and Col-2 (**d**) expression demonstrated fluorescence was decreased in the OA group, while enhancement after treated with calycosin. Groups: sham, OA and 50 mg/kg calycosin treatment groups. Data are presented as the mean ± SD

The animal experimental design is illustrated in Fig. 2a. To evaluate the efficacy of calycosin (PubChem CID: 5,280,448, Fig. 2b), we performed pathological staining of cartilage tissue, including Safranin O/Fast green and toluidine blue. Our results indicate that, in the sham group, sections were uniformly stained. Cartilage surface was smooth and continuous, with oval or round-shaped chondrocytes. The cartilage layer was thick and intact. However, the cartilage thickness and chondrocyte populations in the model group were lower than those in the sham group, which resulted in higher OARSI scores (Fig. 2c, d). It is noteworthy that these phenomena were reversed when treated with calycosin. The calycosin-treated group shows superficial zone restoration, uniform staining, and regular distribution of chondrocytes (Fig. 2d). Col-2 and Sox-9 are the main proteins in cartilage. Immunofluorescence staining showed that Col-2 and Sox-9 were reduced in the OA group but increased in the cartilage matrix after being treated with calycosin (Fig. 3).

### Analysis of PPI Networks

**Fig. 4:**
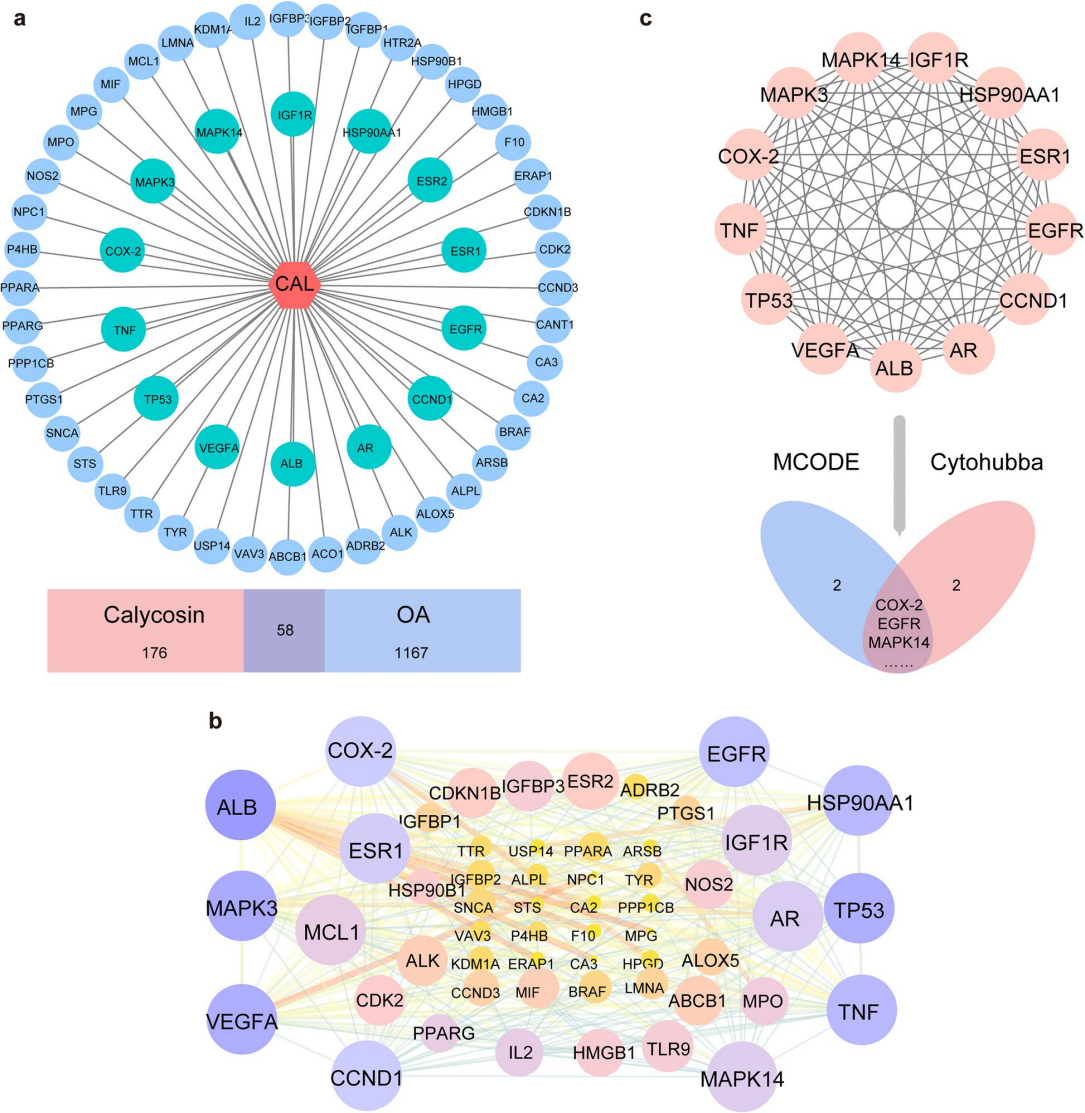
Results of network pharmacology analysis. **a** Intersection gene targets mapping network and Venn diagram show 58 overlapped gene targets. **b** Protein–protein interaction (PPI) network. **c** Network Topology Map of 13 hub targets screened from Cytohubba and MCODE plugins. Network nodes represent proteins, edges represent protein–protein associations, node size means MCODE score, edge size means combined score

Our screening strategy identified 234 calycosin-related and 1225 OA-related targets, respectively. Each predicted target occurs at least two times in different databases. According to the Venn diagram, 58 overlapping genes were identified (Fig. 4a). Protein–protein interaction (PPI) network of 55 nodes (3 isolated nodes are removed), 367 edges were established from the STRING database (confidence > 0.4, medium confidence) (Fig. 4b). To find the hub genes and clusters, the Cytohubba and MCODE plugins in Cytoscape were used. Accordingly, we identified two core clusters (Figure A1a-b, Supplementary figure) and 13 hub genes (Ccnd1, Mapk3, Tp53, Egfr, Hsp90aa1, Ptgs2, Tnf, Esr1, Vegfa, Mapk14, Igf1r, Ar, Alb) (Fig. 4c). Among them, 13 genes overlapped between the two plugins, including COX2, EGFR, and mitogen-activated protein kinase (MAPK14).

### GO and KEGG Pathway Enrichment Analysis

**Fig. 5:**
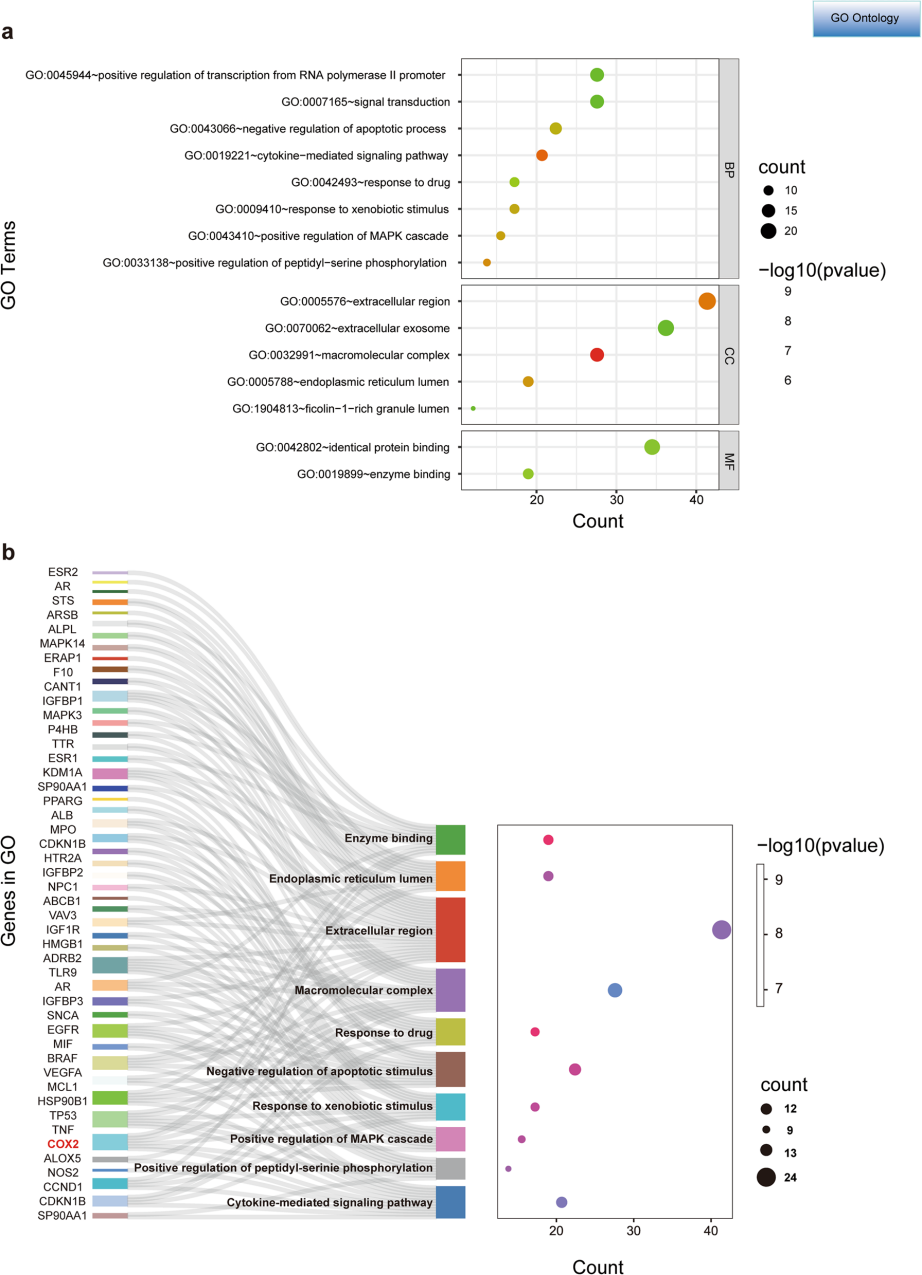
Results of GO enrichment analysis. **a** Top 15 significantly enriched GO functional annotations of intersection genes. **b** Sankey dot plot shows genes involved in the Top 10 GO terms

**Fig. 6:**
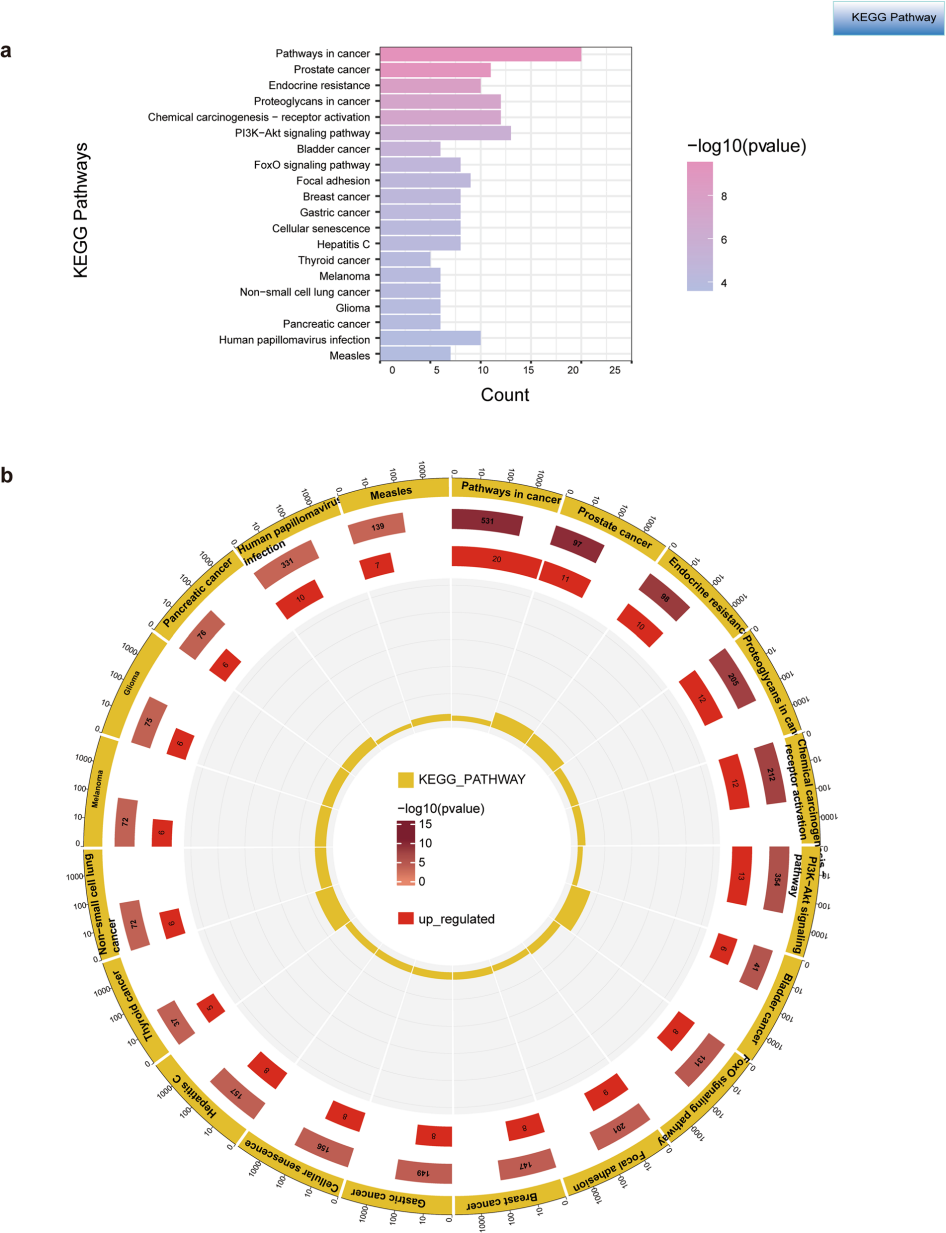
Results of KEGG Pathway enrichment analysis. **a**, **b** Top 20 KEGG pathways with the lowest adjusted *p* values. The bar plot shows the -log10P values for the enriched KEGG pathways and the sub-circle plot shows up-regulated counts and rich factors. The X-axis is the count of the GO terms/pathways, and the Y-axis is the name of the GO terms/pathways. The larger the circle, the greater the number of target genes in the GO terms/pathways. The redder the color, the smaller the -log10 (*p*-value)

We performed GO functional enrichment and KEGG pathway analysis using the DAVID database (a P < 0.01 was considered Statistical significance). The GO terms were classified into three categories: biological process (BP), cellular component (CC); and molecular function (MF). Finally, 372 GO Terms (260 in GO_BP, 45 in GO_CC, and 67 in GO_MF) and 100 KEGG pathways were identified. The top 15 GO functional annotations were presented in Fig. 5a. The categories of GO_BP were response to positive regulation of transcription from RNA polymerase IIpromoter, signal transduction, negative regulation of apoptotic process, cytokine-mediated signaling pathway, response to drug, response to xenobiotic stimulus, positive regulation of MAPK cascade, and positive regulation of peptidyl-serine phosphorylation. The critical GO_CC were related to extracellular region, extracellular exosome, macromolecular complex, endoplasmic reticulum lumen and fcolin-1-rich granule lumen. The critical GO_MF were mainly enriched in the identical protein binding and enzyme binging. The Sankey plot indicates the related genes contained in the top 10 GO terms, in which COX2, TNF, and AR occur six times (Fig. 5b). The KEGG annotations are mainly associated with pathways in cancer, prostate cancer, endocrine resistance, proteoglycans in cancer, chemical carcinogenesis-receptor activation, and the PI3K-Akt signaling pathway, etc. (Fig. 6a, b).

### Molecular Docking

**Fig. 7:**
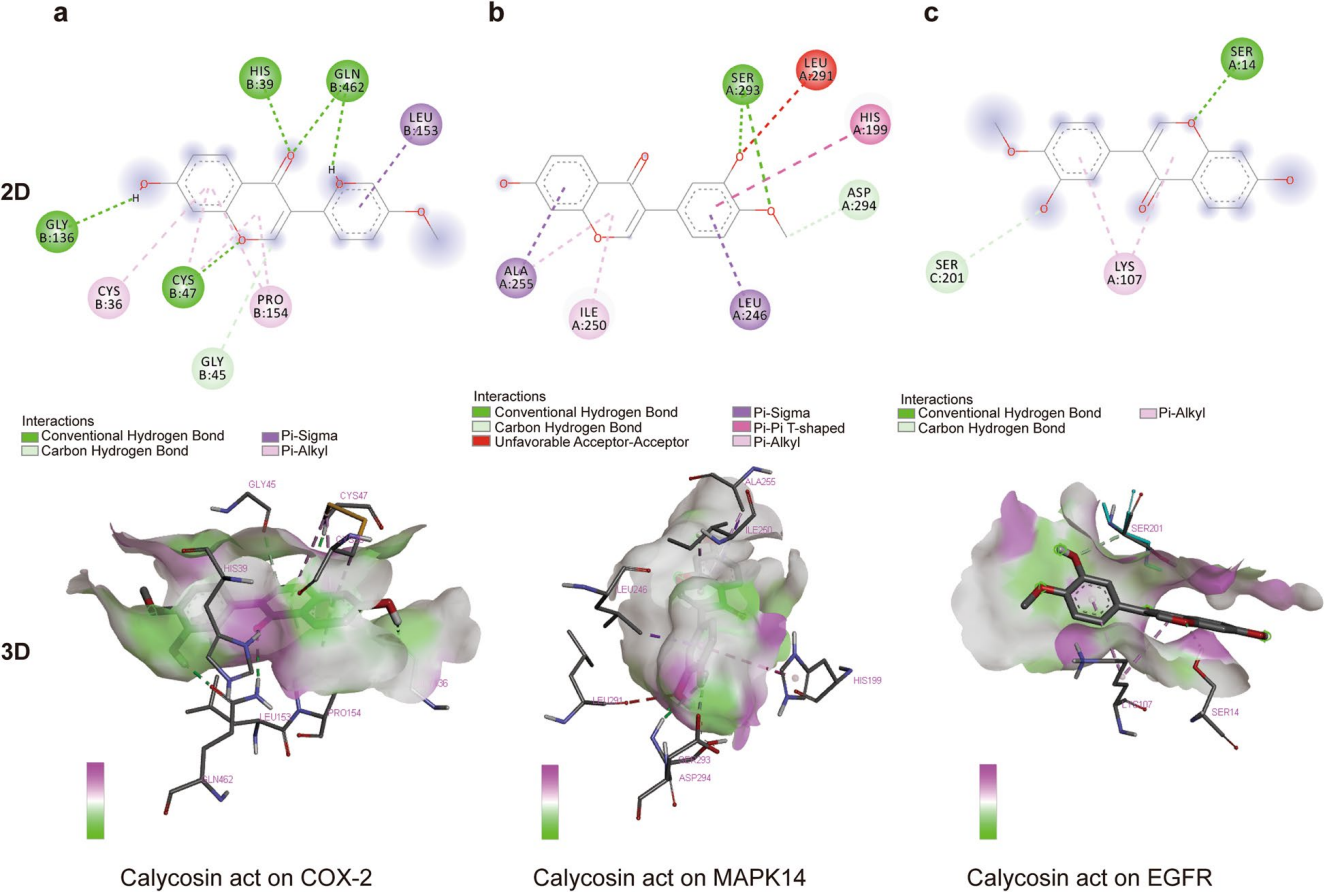
Molecular docking results of calycosin and hub genes. **a** COX-2 (**b**) MAPK14 (**c**) EGFR. Different colors represent the different interactions

The 12 hub targets (ALB was removed) and calycosin were used as receptors and ligand, respectively, for further molecular docking to investigate the affinity between ligand and protein targets. The stability of the binding of the receptors and ligand depends on the binding affinity. The lower the binding affinity, the more stable the binding conformation of the receptor and the ligand. Therefore, we selected the receptor with the lowest affinity that binds to calycosin. The screening results are shown in Table 2, which revealed that COX-2 (Binding sites: GLY45, HIS39, CYS47, CYS36, GLY136, LEN153, GLN452, and PRO154), MAPK14 (Binding sites: SER293, LEU291, HIS199, ASP294, LEU246, ILE250, and ALA255) and EGFR (Binding sites: SER14, SER201, and LYS207) could be representative proteins (Fig. 7). The interactions between calycosin and other potential targets are shown in Supplementary Figure A2. These evidences suggest that calycosin has the ability to co-target COX-2, EGFR and MAPK14.

**Table 2:** Docking results from AutoDock Vina

Hub Targets	PDB ID	Binding Affinity(kcal/mol)
COX-2	5IKQ	-9.5
MAPK14	6SFO	-8.5
EGFR	2XKN	-8.4
IGF1R	1P4O	-8.3
MAPK3	4QTB	-7.9
TP53	3ZME	-7.5
TNF	5MU8	-7.5
ESR1	5ACC	-7.2
AR	3FYW	-6.7
VEGFA	4KZN	-6.6
CCND1	6P8E	-6.5
HSP90AA1	3O01	-6.2

Calycosin alleviates IL-1β induced inflammation in chondrogenic ADTC5 cells.

**Fig. 8:**
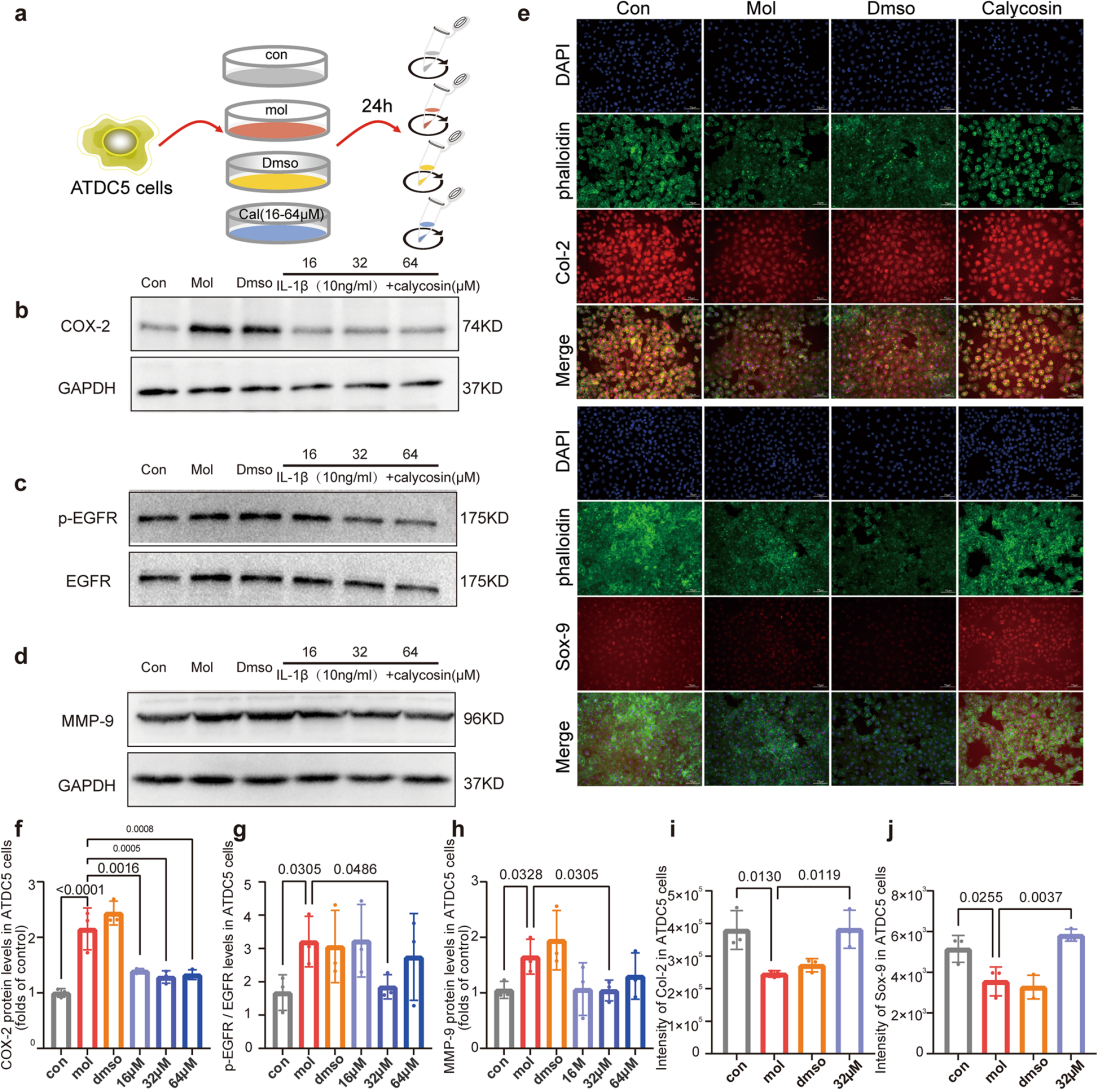
Calycosin inhibits IL-1β induced inflammation in vitro. **a** ADTC5 cells groups and intervention. **e** Representative images of immunofluorescence staining of Col-2 and Sox-9. **b**-**d** Representative western blot bands of COX-2, EGFR and MMP-9 are shown in the CON, MOL, DMSO, and calycosin treatment groups. GAPDH was used as a loading control. The protein levels normalized with GAPDH were shown as fold change relative to the control group. **f**–**h** The quantification analysis of the expression levels of COX-2, p-EGFR and MMP-9. **i**, **j** Quantitative analysis of the Col-2 and Sox-9 immunofluorescence intensity in each group. Data are presented as the mean ± SD

In chondrogenic ADTC5 cells, the proinflammatory cytokine IL-1β is markedly overexpressed in OA and mimics the chondrocyte inflammation model. ADTC5 cells were exposed to IL-1β (10 ng/mL), which caused a considerable decrease in COX-2 and phospho-EGFR (p-EGFR) expression (Fig. 8a-c), while no significant changes of MAPK14 or p-MAPK14 (Figure A1, Supplementary figure) were observed. In addition, compared to the OA group, ADTC5 chondrogenic cells showed a moderate decrease of MMP-9 when co-cultured with 32 μM calycosin (Fig. 8d). In agreement with in vivo experiments, immunofluorescence staining (Fig. 8e) demonstrated that Col-2 and Sox-9 were remarkably increased after calycosin treatment. The quantification analysis results were shown in Fig. 8 f-j.

## Discussion

The prevalence of OA is increasing worldwide due to population aging. Cartilage degradation and chondrocyte damage play an important role in the pathological mechanism of OA. Calycosin (PubChem CID: 5,280,448) is a typical active ingredient extracted from traditional Chinese medicine, but its mechanisms are unclear. We therefore explored the potential targets and underlying mechanisms of calycosin in the treatment of OA through network pharmacology and experimental validations. The present study suggests that calycosin contributes to cartilage repair, possibly associated with promoting Col-2 and Sox-9 levels in vitro and in vivo. In addition, calycosin can attenuate IL-1β-induced inflammation by inhibiting COX-2, p-EGFR, and MMP-9 expression in chondrogenic ADTC5 cells, thereby alleviating cartilage matrix degradation. These data indicate that calycosin improves chondrocyte inflammatory damage, attenuates cartilage degradation and is expected to be a potential drug candidate for OA treatment.

According to the experiments and analysis of the hub genes and main significant KEGG pathways, the potential mechanisms of calycosin treatment of OA could be attributed to the following aspects.

### Improve cartilage synthesis and catabolic imbalance

OA is a typical chronic inflammatory disease, and there is still no drug that can prevent the degenerative cascade reaction. With the aggravation of OA, the cracks on the surface of the cartilage deepened, ulcers formed, and gradually developed into deep cartilage tissue [65]. This is closely related to an imbalance between chondrocyte synthesis and catabolism, including excessive degradation of structural proteins such as Col-2 and increased synthesis of MMPs [66, 67]. Currently, the first-line drugs approved by the FDA are mainly NSAIDs, among which the COX-2 inhibitors can effectively relieve the pain. However, both traditional NSAIDs and selective COX-2 inhibitors have a certain degree of adverse reactions, which results in limited clinical application. Calycosin has been demonstrated to have various pharmacological activities. It especially exhibits a strong anti-inflammatory effect. On the one hand, since low-grade inflammation plays a critical role in the pathogenesis of OA, calycosin can inhibit inflammatory factors such as COX-2 to relieve OA. On the other hand, MMPs play a crucial role in the destruction of cartilage, calycosin decreases the MMP-9 produced by chondrocytes [66, 68]. After being treated with calycosin, the expressions of Col-2 and Sox-9 are also increased. This contributes to cartilage collagen synthesis. Mice lacking EGFR in cartilage (Col2-Cre) exhibited reduced chondrocyte and synovial fluid secretion compared to their wild-type siblings [67], demonstrating that EGFR is essential for maintaining the homeostasis of articular cartilage. However, like some other growth factor signaling pathways, EGFR plays a dual role in articular cartilage. On the one hand, EGFR signaling can promote articular surface lubrication by increasing proteoglycan 4 (PRG4) [69]. On the other hand, EGFR signaling also plays a catabolic action by inhibiting the chondrogenic master transcription factor Sox-9 and stimulating MMP9 expression [70]. Therefore, further study is needed to determine the specific role that EGFR signaling plays in the OA process. Though calycosin can decrease the phosphorylation levels of SRC, EGFR, ERK1/2, and Akt in breast cancer lines and meningitis [63, 71], its role in chondrocytes remains unclear. Our results show that the binding capacity of calycosin with EGFR is consistent with previous studies [71, 72]. In this study, we report the beneficial aspects of inhibiting EGFR, the effects of calycosin did not cause negative impacts. This phenomenon may be due to the various biological activities of calycosin. We have only discussed the anti-inflammatory effects here. Other.

pharmacological effects of OA are still worth exploring. For example, calycosin has been reported to have a strong osteogenic activity, it can inhibit bone resorption and stimulate bone formation [73, 74]. Another study shows that calycosin plays an anti-osteoporosis effect through the IGF1R/PI3K/Akt signaling pathway [30]. In addition, calycosin exerts estrogenic properties, which are also controversial in OA, probably related to interaction with EGFR. Therefore, the various physiological activities and mechanisms of calycosin need to be further studied.

### Other possible anti-OA mechanisms

Our findings provide insights into the relationship between OA and calycosin, the hub genes, and significant GO and KEGG pathways, which deserve further study.

Within hub genes, vascular endothelial growth factor A (VEGFA) has been shown to be involved in the OA process [75]. Increased expression of VEGFA was positively correlated with the secretion of ECM, including collagen II and aggrecan [76]. In addition, age and gender occupied an unignored position among the well-established epidemiological risk factors of OA [1, 77]. This phenomenon has attracted the attention of researchers who are interested in the role of estrogen in OA [78]. An investigation of OA combining transcriptomic and proteomic characterization data revealed that the estrogen receptor agonists diethylstilbestrol and alpha-estradiol hold promise as therapeutic candidates and drug targets [79]. Calycosin, is a member of 7-hydroxyisoflavones and a member of 4'-methoxyisoflavones, showing a similar structure to estrogen estradiol and acts as a plant-derived phytoestrogen, it is functionally related to an isoflavone. In addition, it possesses estrogen-like activity [36]. Our results also indicate that calycosin has the capacity to bind with estrogen receptors ESR1, which makes it widely studied in cancers like breast cancer. Therefore, seeking alternative solutions to estrogen replacement therapy may benefit from phytoestrogen therapy due to its fewer side effects [80, 81]. In the present study, the phosphoinositide-3-kinase (PI3K)/Akt signaling pathway was significantly enriched. The PI3K-Akt signaling pathway plays a crucial role in OA, as does the mitogen-activated protein kinase (MAPK) signaling pathway. Studies have reported that the increased expression level of P-Akt can induce chondrocyte proliferation [82] and inhibition of phosphorylation of MAPK and NF-κB can inhibit the expression of COX-2, MMPs, iNOS in chondrocytes [83, 84]. It is reported that calycosin induces apoptosis in osteosarcoma cell lines via ERβ-mediated PI3K/Akt signaling pathways. Recent studies have shown that NF-κB and PI3K/AKT are inhibited by calycosin in OA chondrocytes, suggesting that it may act as a protective agent against OA. [85]. The pathways and genes enriched in this paper are still valuable to be explored. Nevertheless, this paper does not verify the intersection targets involved one by one, it only provides a map of possible targets, which is the limitation of our study.

In this paper, we explored the possible targets of calycosin in the treatment of OA through bioinformatics mining. Furthermore, experiments indicated that calycosin has therapeutic potential by targeting COX-2/EGFR. However, further research is needed before calycosin can be considered a therapeutic option for OA. In summary, by integrating network pharmacology and molecular experiments, we proposed a map of possible targets and a relatively low-risk approach to OA. These findings provide strong support for calycosin as a potential therapeutic, in response to the global clinical challenge of OA.

## Conclusions

Based on the above experimental results, we constructed a possible mechanism for calycosin in the treatment of OA. That is, calycosin can inhibit the COX-2/EGFR signaling pathways and exert powerful anti-inflammatory properties to promote cartilage repair. Moreover, it up-regulated Col-2 and Sox-9 levels in vitro and in vivo, thus rescuing chondrocyte synthesis and catabolism imbalance.

### Supplementary Information

**Additional file 1: Figure A1. **(a-b) Cluster analysis of the intersection targets show 2 core clustering graphs from PPI network. (c-d) Representative western blot bands and quantification analysis of the p-MAPK14. **Figure A2.** Molecular docking results of calycosin and hub genes. (a) MAPK3 (b) TP53 (c) TNF (d) ESR1 (e) IGF1R (f) AR (j) VEGFA (h) CCND1 (i) HSP90AA1.

## Data Availability

The data and materials of this study are available from the corresponding author upon reasonable request. The datasets generated and/or analyzed during the current study are available in the Traditional Chinese Medicine Systems Pharmacology repository, http://lsp.nwu.edu.cn/tcmsp.php.

## Abbreviations

OA: Osteoarthritis
Col-2: Type II collagen
Sox-9: SRY-Box Transcription Factor 9
COX-2: Activate cycloxygenase-2
MMPs: Matrix metalloproteinases
TNF-α: Tumor necrosis factor- α
IL: Interleukin;
EGFR: Epidermal growth factor receptor
TGFα: Transforming growth factor alpha
HB-EGF: Heparin-binding epidermal growth factor-like growth factor
GWAS: Genome-wide association studies
NSAIDs: Anti-inflammatory drugs
TCM: Traditional Chinese medicine
SEA: Similarity Ensemble Approach
TCMSP: Traditional Chinese Medicine Systems Pharmacology
TTD: Therapeutic target database
OMIM: Online Mendelian Inheritance in Man
STRING: Search Tool for Recurring Instances of Neighboring Genes
PPI: Protein–protein interaction
MCODE: Molecular Complex Detection
GO: Gene Ontology
KEGG: Kyoto Encyclopedia of Genes and Genomes
DAVID: The Database for Annotation, Visualization, and Integrated Discovery
ACLT: Anterior cruciate ligament transected
OARSI: Osteoarthritis research society international
DMEM/F-12: Dulbecco's Modified Eagle Medium/Nutrient Mixture F-12
CON: Control
MOL: Model
CAL: Calycosin
PBS: Phosphate-buffered saline
ANOVA: Analysis of variance
BP: Biological process
CC: Cellular component
MF: Molecular function
