# Supplementary material for: Calycosin ameliorates osteoarthritis by regulating the imbalance between chondrocyte synthesis and catabolism
Source: BMC Complement Med Ther. 2024 Jan 22;24:48. doi: 10.1186/s12906-023-04314-z (PMC10804771; doi:10.1186/s12906-023-04314-z)
Supplement: Supplementary file 1 — Additional file 1: Figure A1. (a-b) Cluster analysis of the intersection targets show 2 core clustering graphs from PPI network. (c-d) Representative western blot bands and quantification analysis of the p-MAPK14. Figure A2. Molecular docking results of calycosin and hub genes. (a) MAPK3 (b) TP53 (c) TNF (d) ESR1 (e) IGF1R (f) AR (j) VEGFA (h) CCND1 (i) HSP90AA1. [file 12906_2023_4314_MOESM1_ESM.pdf]

Appendix A

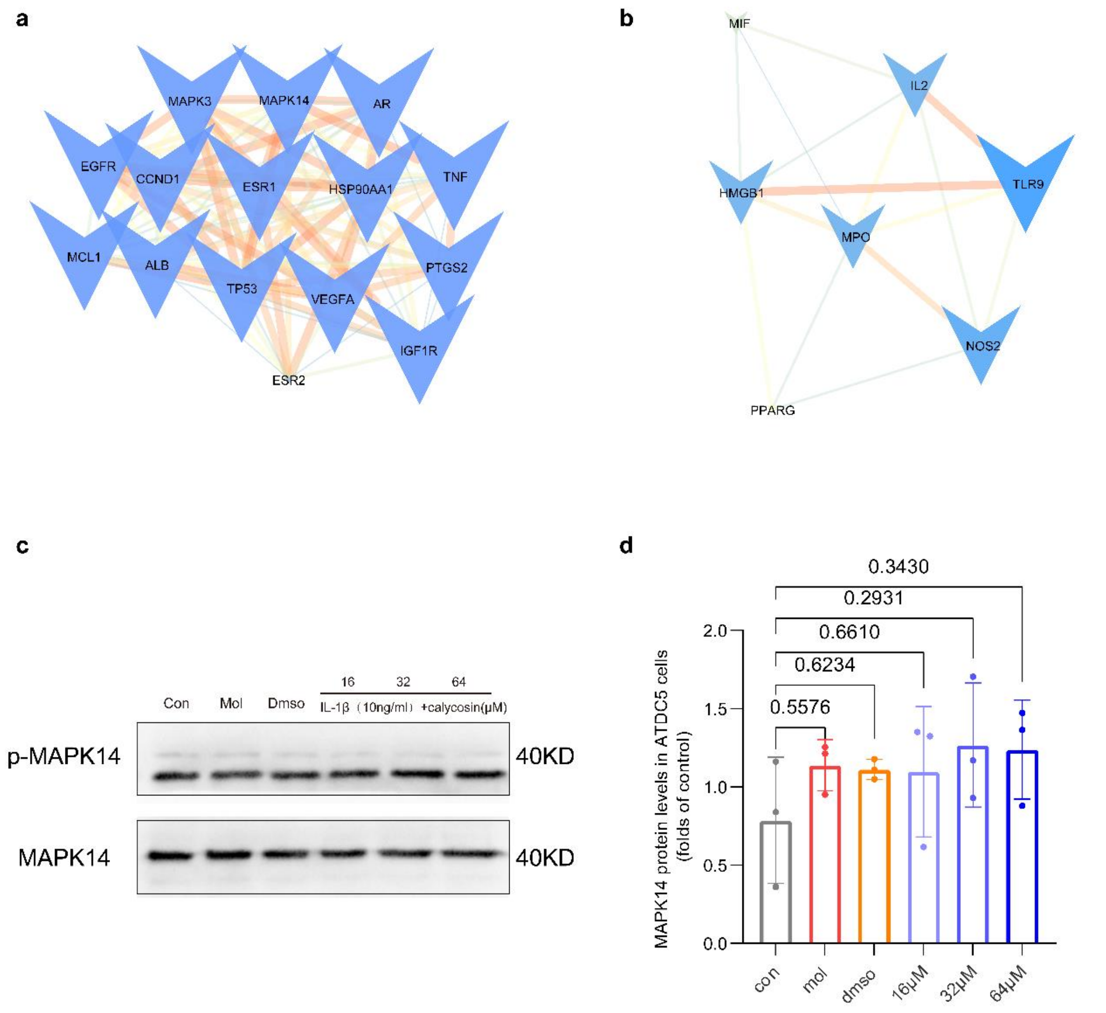

Figure A1. (a-b) Cluster analysis of the intersection targets show 2 core clustering graphs from PPI network. (c-d) Representative western blot bands and quantification analysis of the p-MAPK14.

Appendix B

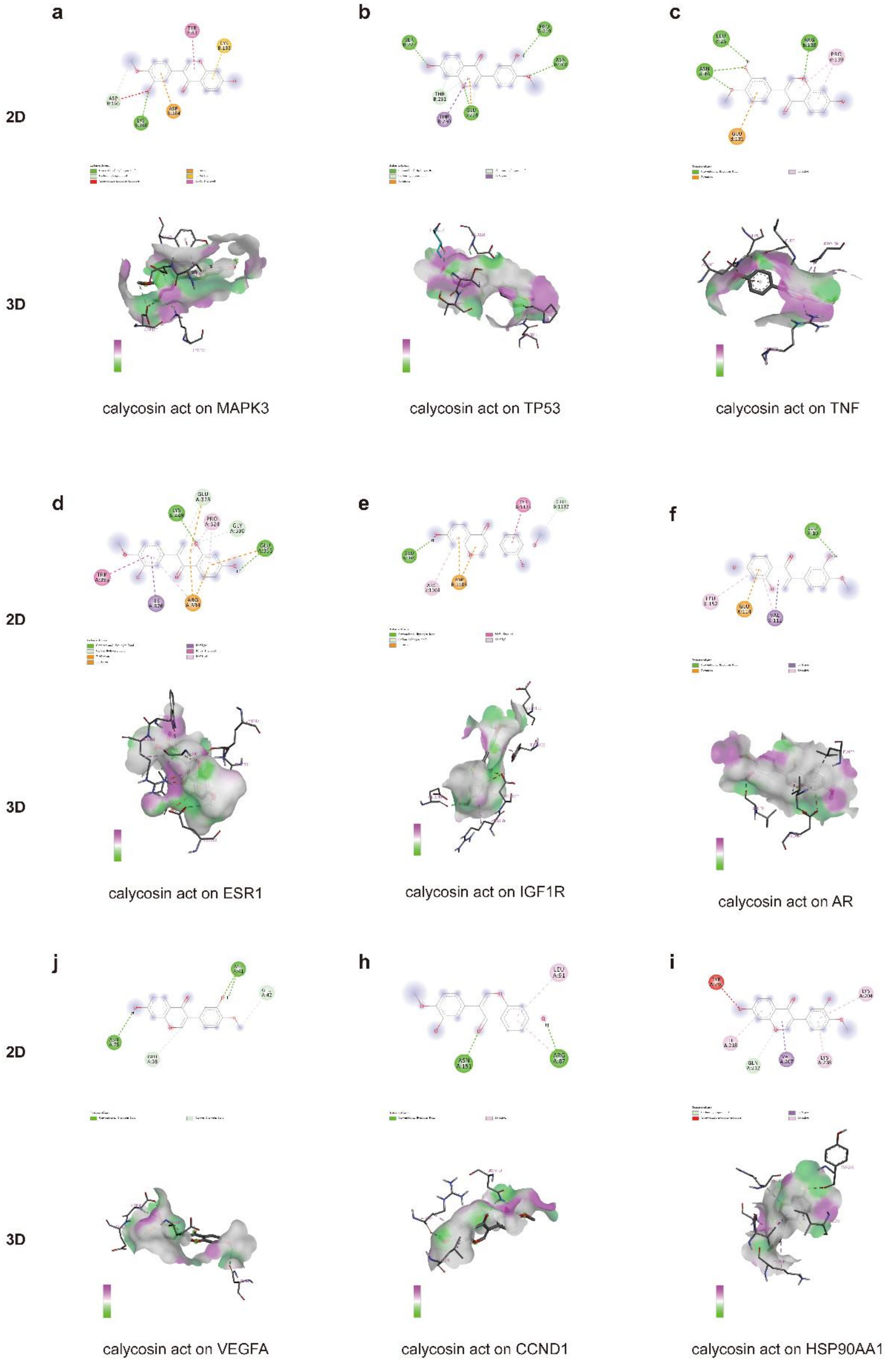

Figure A2. Molecular docking results of calycosin and hub genes. (a) MAPK3 (b) TP53 (c) TNF (d) ESR1 (e) IGF1R (f) AR (j) VEGFA (h) CCND1 (i) HSP90AA1

Appendix C

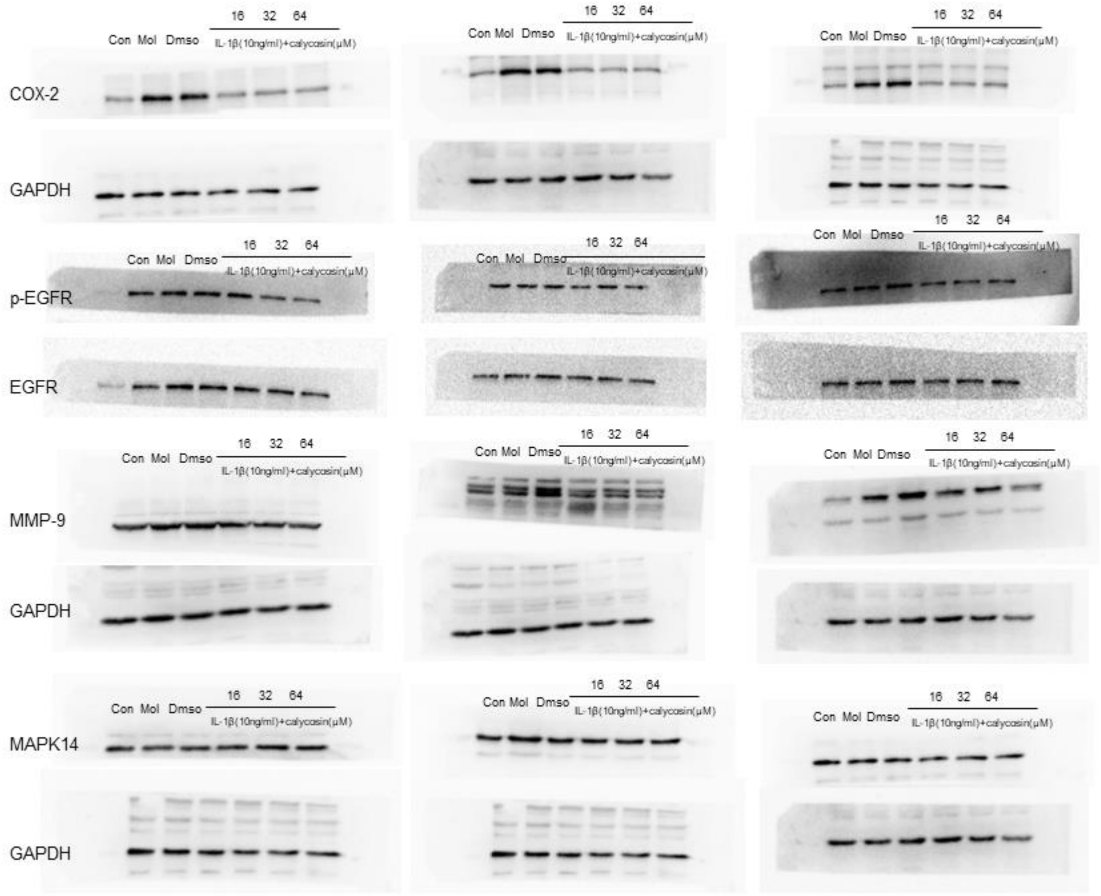

Figure A3. Western Blotting.
